# Accidentally discovered non‐communicating membranous ventricular septal aneurysm in a middle‐aged male patient

**DOI:** 10.1002/ccr3.7642

**Published:** 2023-07-17

**Authors:** Mahmoud Abdelnabi, Abdallah Almaghraby, Yehia Saleh, Juthipong Benjanuwattra, Hoda Abdelgawad

**Affiliations:** ^1^ Department of Internal Medicine Texas Tech University Health Sciences Center Lubbock Texas USA; ^2^ Department of Cardiology Ibrahim Bin Hamad Obaidullah Ras Al Khaimah UAE; ^3^ Department of Cardiology Houston Methodist DeBakey Cardiology Associates Houston Texas USA; ^4^ Department of Cardiology, Faculty of Medicine Alexandria University Alexandria Egypt; ^5^ Department of Cardiology King's College Hospital NHS Trust London UK

**Keywords:** cardiac computed tomography, congenital heart disease, echocardiography, membranous ventricular septal aneurysm, multimodality cardiac imaging

## Abstract

Membranous interventricular septal (MIVS) aneurysm is a rare often asymptomatic, accidentally discovered congenital anomaly, which might be complicated with right ventricular obstruction, rupture, thromboembolism, and conduction abnormalities.

## Graphical abstract

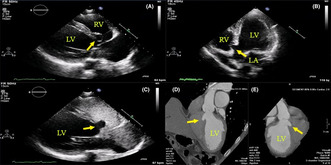

## 1 CASE PRESENTATION

**FIGURE 1:**
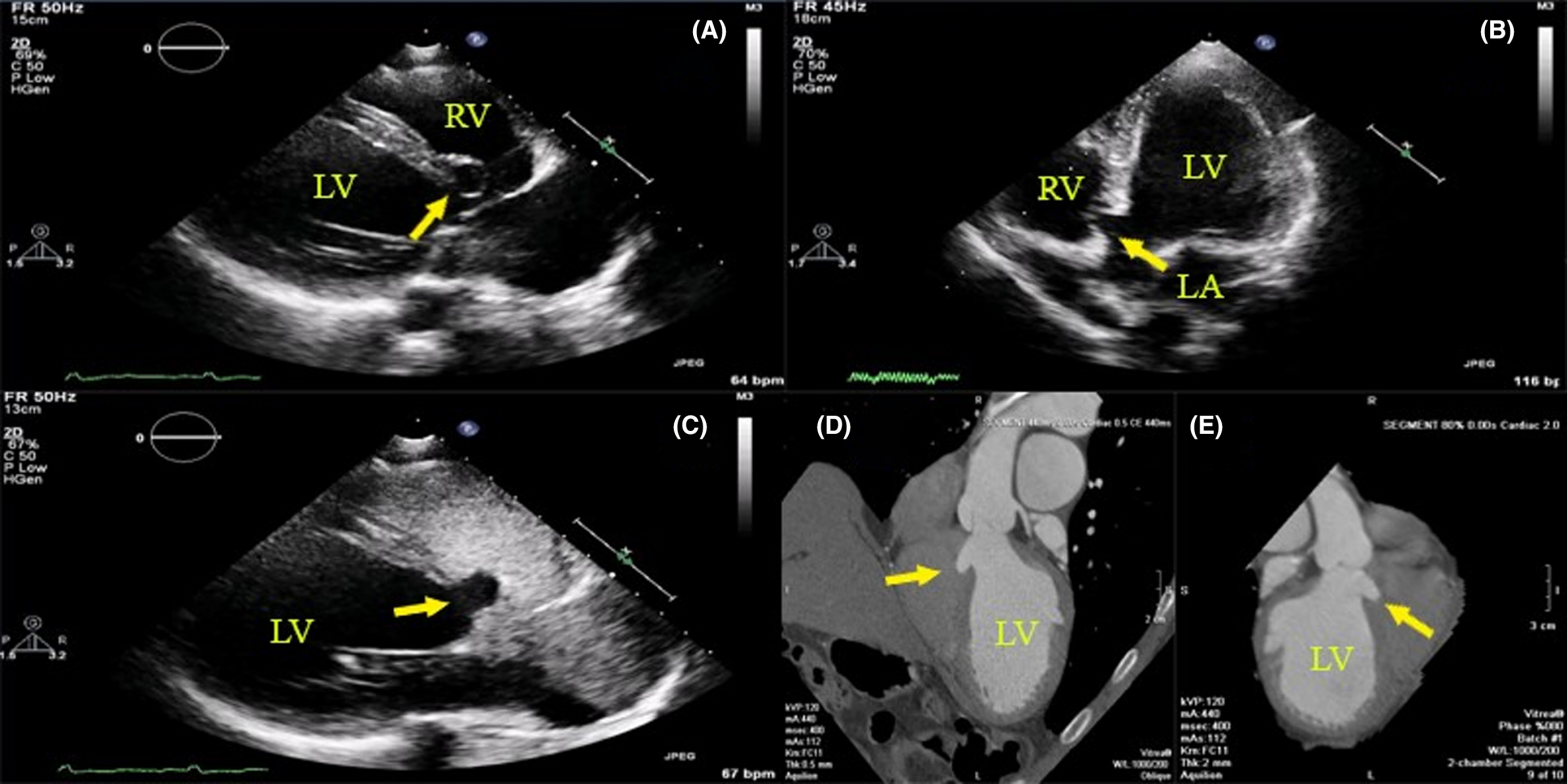
(A–C) 2D TTE showed an incidentally discovered MIVS aneurysm with no left to right shunt by agitated saline. (D, E) Cardiac MDCT confirmed the presence of a cauliflower‐shaped MIVS aneurysm.

A man in his late 50s with no previous past medical history presented with a 6‐month history of worsening dyspnea with no orthopnea or paroxysmal nocturnal dyspnea (PND). On cardiac examination, he had a loud pansystolic systolic murmur over the apex radiating to the axilla. His electrocardiogram (ECG) showed sinus rhythm with interventricular conduction delay while his chest X‐ray showed marked cardiomegaly. Transthoracic echocardiography (TTE) showed a reduced global left ventricular (LV) systolic function with an estimated left ventricular ejection fraction of 30%–35% with moderate mitral regurgitation and incidentally discovered membranous part of the interventricular septum (MIVS) aneurysm without detectable left to right shunt by agitated saline (Figure 1A–C). Cardiac multidetector computed tomography (MDCT) confirmed the presence of a cauliflower‐shaped aneurysm at MIVS with no coronary artery disease detected. (Figure 1D,E). Per his request, no further invasive work‐up was done and he was started on heart failure guideline‐directed medical therapy and scheduled for close outpatient follow‐up.

Membranous part of the interventricular septum aneurysm is a rare congenital abnormality that is incidentally discovered in most cases.^1^ It has been associated with ventricular septal defects and outflow abnormalities, such as transposition of the great vessels.^1^ Patients are usually asymptomatic, but they might present with complications such as right ventricular obstruction, rupture, thromboembolism, and conduction defects.^2^ It should be differentiated from anatomically‐related aneurysms and aneurysmal‐like structures arising in and around the left ventricular outflow tract such as sinus of Valsalva aneurysms.^2^ The diagnosis of MIVS aneurysm is often made by TTE; however, multimodality cardiac imaging such as cardiac MDCT and cardiac magnetic resonance imaging (CMR) may be required for the diagnosis of associated anomalies and complications.^3^ Conservative treatment and close follow‐up are the cornerstone of management in uncomplicated cases, while surgical resection is indicated if there is concomitant heart disease, hemodynamic abnormalities, or complications.^2^

## AUTHOR CONTRIBUTIONS

**Mahmoud Abdelnabi:** Resources; writing – original draft; writing – review and editing. **Abdallah Almaghraby:** Writing – review and editing. **Yehia Saleh:** Writing – review and editing. **Juthipong Benjanuwattra:** Writing – review and editing. **Hoda Abdelgawad:** Supervision; writing – review and editing.

## CONFLICT OF INTEREST STATEMENT

None declared.

## PATIENT CONSENT STATEMENT

The authors have obtained written informed consent from the patient to publish her medical history/course and case details in accordance with the journal's patient consent policy.

## Data Availability

All case‐related data are available as part of the article and no additional source data are required.
